# A hyper-matheuristic approach for solving mixed integer linear optimization models in the context of data envelopment analysis

**DOI:** 10.7717/peerj-cs.828

**Published:** 2022-01-20

**Authors:** Martin Gonzalez, Jose J. López-Espín, Juan Aparicio, El-Ghazali Talbi

**Affiliations:** 1 Center of Operations Research, Universidad Miguel Hernández de Elche, Elche, Spain; 2 INRIA, Université des Sciences et Technologies de Lille (Lille I), Lille, France

**Keywords:** Hyper-matheuristic, Metaheuristics, Exact methods, Mixed integer problems, MILP decomposition, Mathematical optimization

## Abstract

Mixed Integer Linear Programs (MILPs) are usually NP-hard mathematical programming problems, which present difficulties to obtain optimal solutions in a reasonable time for large scale models. Nowadays, metaheuristics are one of the potential tools for solving this type of problems in any context. In this paper, we focus our attention on MILPs in the specific framework of Data Envelopment Analysis (DEA), where the determination of a score of technical efficiency of a set of Decision Making Units (DMUs) is one of the main objectives. In particular, we propose a new hyper-matheuristic grounded on a MILP-based decomposition in which the optimization problem is divided into two hierarchical subproblems. The new approach decomposes the model into discrete and continuous variables, treating each subproblem through different optimization methods. In particular, metaheuristics are used for dealing with the discrete variables, whereas exact methods are used for the set of continuous variables. The metaheuristics use an indirect representation that encodes an incomplete solution for the problem, whereas the exact method is applied to decode the solution and generate a complete solution. The experimental results, based on simulated data in the context of Data Envelopment Analysis, show that the solutions obtained through the new approach outperform those found by solving the problem globally using a metaheuristic method. Finally, regarding the new hyper-matheuristic scheme, the best algorithm selection is found for a set of cooperative metaheuristics ans exact optimization algorithms.

## Introduction

Mixed Integer Linear Programs (MILPs) address mathematical optimization problems involving two families of variables: discrete and continuous ones. Both the objective function as well as the constraints are linear. This family of optimization problems appears for many real-life applications in various domains. Indeed, many real problems can be formulated using MILP models, for example: packing, knapsack, inventory, production planning, location, resource allocation, routing and scheduling problems, to name but a few (Winston & Goldberg, 2004). This large applicability has led to an increased interest in the development of efficient algorithms for solving this general and popular class of optimization problems.

MILP models are generally NP-hard problems. Approximation algorithms have been developed in response to the impossibility of solving a great variety of important optimization problems. Very often, one is confronted with the fact that the problem is NP-hard, making it really difficult to obtain an optimal solution in a reasonable time (Hochba, 1997). For all intents and purposes, we use two families of algorithms to solve MILPs: exact algorithms and heuristics. The exact methods (*e.g*. branch and bound, branch and cut, branch and price) are generally applicable, but they have been proven to be laborious for large or more complex problems. When instances become too large or difficult for exact methods, heuristics and particularly metaheuristics are often used. A metaheuristic is a high-level procedure to select among different heuristics. We can examine two types of metaheuristics: single solution algorithms (*e.g*. local search, tabu search) and algorithms based on population (*e.g*. evolutionary algorithms, swarm optimization) (Talbi, 2009). Metaheuristics do not, however, generally guarantee that the best solutions are found. Thus, the combination of metaheuristics and exact optimization algorithms can offer a more efficient and effective resolution method (Talbi, 2016). A general classification of this hybridization is discussed by Jourdan, Basseur & Talbi (2009), and some examples can be found in the literature (Pradenas et al., 2013; Li et al., 2012).

In this paper, a new hyper-matheuristic methodology, based on the matheuristic previously introduced in González et al. (2017), is developed to find solutions for MILP models in the context of Data Envelopment Analysis (DEA) (Vanderbeck & Wolsey, 2010). Nowadays, DEA is one of the most used non-parametric techniques in Economics and Engineering to measure technical efficiency from a data sample of firms. Regarding our methodology, the matheuristic allows for MILP-based decomposition, where the main problem is broken down into two hierarchical subproblems, since it is easier to solve them separately using distinct categories of optimization algorithms (Raidl, 2015). This breakdown is based on the characteristics of the continuous and discrete decision variables. The hyper-matheuristic methodology is proposed from this matheuristic. The hyper-heuristic concept (Pillay & Banzhaf, 2009) has been applied in the context of hybrid heuristics (Wang et al., 2019; Li et al., 2020) to find the best combination of heuristics. In this work, we propose a generalization of the hyper-heuristic methodology for matheuristics that combines exact algorithms and metaheuristics, which is called the hyper-matheuristic approach.

The aim of the proposed hyper-matheuristic methodology is to find the best combination between metaheuristics and exact methods for a given type of MILP models within the framework of DEA. For this, some input instances of the problem are evaluated with several iterations of the algorithm, training the hyper-matheuristic and obtaining a good solution for any problem. Within the hyper-matheuristic, several parameters have been established to generate many different metaheuristics. The functionalities of the generated metaheuristics, which depend on the values of these parameters, where metaheuristics like Evolution Algorithm (EA) (Holland, 1973), Scatter Search (SS) (Glover, Laguna & Marti, 2003), Tabu Search (TS) (Glover, 1997) or Greedy Randomized Adaptive Search Procedure (GRASP) (Resende & Ribeiro, 2003) can be generated automatically. All of these parameters take different values. These values are studied in the experiments, having set certain limit values, and they represent inputs for the algorithm.

The matheuristic method was designed by examining the various synergies between metaheuristics and exact methods, in order to find the best combination for resolving MILP problem. A list of existing approaches combining exact methods and metaheuristics for MILP optimization can be found in Puchinger & Raidl (2005):

- Collaborative combinations: self-contained optimization algorithms exchange information extracted from the search. The different algorithms are independent. There is no direct relationship to the internal workings of the algorithms. Exact method and heuristic algorithms can be executed sequentially, interwoven or in parallel.
- Integrative combinations: in these types of algorithms, hybridization addresses the functional composition of a single optimization method. A given internal function of an optimization algorithm is replaced by another optimization algorithm.

The matheuristic algorithm (González et al., 2017) used in this paper for the proposed hyper-matheuristic employs an integrative combination, where by the metaheuristic supplies information to the exact method, which solves the problem and returns some new information to the metaheuristic. The basic concept is to break the problem down into much smaller subproblems which can be accurately solved using cutting-edge mathematical programming algorithms. The variables and the constraints are divided up into two sets, which break the main problem down into two hierarchical subproblems: the metaheuristic determines the decision variables in one set and the exact method optimizes the problem in the other. In the literature, there are some works where certain exact techniques are improved using approximation techniques (metaheuristics), as in the case of Poojari & Beasley (2009), where Bender’s decomposition is optimized through a genetic algorithm, integrating the latter as a seed generator for decomposition.

Moreover, a hyper-metaheuristic scheme has been included in the proposed methodology for an autonomous design of metaheuristics. Certain design parameters define the characteristics of each metaheuristic, and these are framed into different search components: Initialize, Improvement, Selection, Combination and Stopping Criteria. In this work, the hyper-metaheuristic methodology (González et al., 2017) has been generalized to matheuristics, in which exact optimization is combined with a set of metaheuristics.

The main contributions in this paper are based on the development of a general methodology in terms of optimization algorithms, being capable of solving MILP problems in the context of DEA. A MILP-based decomposition is studied that combines metaheuristics and exact methods in a single algorithm called a matheuristic. A final algorithm is implemented to obtain the best combination of those algorithms previously mentioned, called hyper-matheuristic.

The paper is organized as follows: In “MILP-Based Decomposition”, we present the proposed breakdown of MILP problems. In “Matheuristic Methodology” we detail the matheuristic strategy that combines linear continuous programming and discrete metaheuristics. “Hyper-Matheuristic Methodology”, focuses on the hyper-matheuristic methodology in which an automatic design of optimization algorithms is carried out. In “Experimental Results”, we provide some computational experiments on a MILP problem. Finally, in “Conclusions and Future Works”, we conclude and point out some future works.

## Milp-based decomposition

In this section, we will handle general notions within the field of MILP models and the developments will be as general as possible. Nevertheless, our approach will be exclusively tuned and tested with problems from Data Envelopment Analysis in “Experimental Results”. We are aware that the technique could be used with other types of MILP models. However, we cannot guarantee the validity of the new approach in those cases. In this respect, further research in this line would be necessary. Let us consider the following linear problem (LP) (1):

(1) $$\matrix{ {\max \left\{ {{cx}:{Ax} \le b,x \ge {\rm 0},{x} \in {{ {R}}^n}} \right\}} \cr }$$

where ***A*** is a *m* × *n* matrix, ***c*** a *n*-dimensional row vector, ***b*** a *m*-dimensional column vector, and ***x*** a *n*-dimensional column vector of continuous variables. If we add the constraint that certain variables must take integer values, we have a MILP (2), that can be written as:

(2) $$\matrix{
   {\max {\bf{cx}} + {\bf{hy}}}  \cr 
   {{\bf{Ax}} + {\bf{Gy}} \le {\bf{b}}}  \cr 
   {{\bf{x}} \ge {\bf{0}},{\bf{x}} \in {R^{\bf{n}}}}  \cr 
   {{\bf{y}} \ge {\bf{0}},{\bf{y}} \in {Z^{\bf{p}}}}  \cr 

 } $$

where ***A*** is again a *m* × *n* matrix, ***G*** is *m* × *p* matrix, ***h*** is a *p* row-vector, and ***y*** is a *p* column-vector of integer variables.

An MILP problem is defined as one where discrete (*y*) variables, which are restricted to integer values, and continuous variables (*x*), and which can be assigned any value on a given continuous interval, are combined with integrality constraints. The integrality constraints allow MILP models to capture the discrete nature of some decisions. For example, a binary variable can be used to decide whether or not any action needs to be taken.

Using MILP solvers to resolve large-scale and complex instances is inefficient in terms of the time spent determining the solution to the problem. Indeed, large MILPs are often difficult to resolve using exact methods, owing to the complexity of the combinatorial nature of the discrete part of the problem. One way to solve large MILPs is to break them down into smaller subproblems, so they can be solved individually. Problem decomposition techniques comprise an approach that is particularly aimed at solving very large and difficult problems. The basic idea is to solve such a large problem by solving a set of smaller problems, combining their solutions to obtain an optimal one for the main MILP problem. For each subproblem, if the optimality criterion is satisfied, the current feasible solution is judged to be the optimal solution of the original MILP problem. If the optimality criterion is not satisfied, other values for the variable in the subproblems are assumed, and the procedure is repeated (Yokoyama & Ito, 2000).

### Popular decomposition techniques

The objective of decomposition techniques is to tackle large-scale problems which cannot be solved by exact optimization algorithms such as MIP solvers (Ralphs & Gelati, 2010). From the integer programming point of view, there are two types of decomposition approaches that exploit the problem structure: constraint decomposition and variable decomposition (Vanderbeck & Wolsey, 2010).

In constraint decomposition techniques, a compact problem is created by the insertion of constraints to obtain a better approximation by eliminating a part of the feasible space that does not contain integer solutions. Outer approximation (cutting plane methods) (Kelley, 1960) or inner approximation (Dantzig-Wolfe method) (Vanderbeck, 2000; Ruszczynski, 1989) are the most popular ones.

In variable decomposition techniques, the decision variables of the problem are generally separated into two groups and the problem is solved in two steps. Bender’s decomposition represents one of the most popular variable decomposition approaches for solving integer programming problems (Bender’s, 1962), being less popular than branch-and-cut, but really common in the literature. This decomposition technique is based on a cycle of two steps in each iteration. At the first step, a subset of integer variables is selected and their values are found. Then, the second step finds an optimal solution for the rest of the continuous variables according to the values allocated to the first subset of variables. In each iteration, some constraints are modified in the sub-problems to improve the solution. This approach has been applied to many problems such as routing, scheduling, network design and planning (Rahmaniani et al., 2017).

From the metaheuristic point of view, one can use either a variable or data decomposition by using some problem features. In variable decomposition techniques, the problem is decomposed into subproblems of similar size following a variable decomposition (for example, time decomposition in scheduling problems). After the resolution of the subproblems, the global solution is constructed from the sub-optimal partial solutions obtained. The subproblems can be solved in independent or hierarchical ways. In data decomposition techniques, the input data (*e.g*. geographical space) of the general problem is divided into different partitions. Then, the problem is solved using all the partitions and the final solutions are aggregated from the sub-solutions obtained from the different partitions. For instance, some clustering algorithms can be applied to partition a geographical space into different regions for routing problems (Reimann, Doerner & Hartl, 2004; Taillard, 1993).

### Variable-based decomposition of MILPs

The main drawback of data-based decomposition is its specificity to the target optimization problem. In our work, a more general decomposition predicated on the type of variables (discrete *vs* continuous) and the complexity of the generated subproblems is carried out. The main problem is decomposed into two different hierarchical subproblems, following the principles of indirect encoding (Talbi, 2009). The master problem is associated to the discrete variables. A solution is encoded using an incomplete solution for the problem in which only the discrete variables are handled. For each solution of the master problem, the subproblem will fix the continuous variables of the solution. It can be seen as decoding the incomplete solution given by the master problem to generate a complete solution for the problem. Then, the constraints associated to the optimization problem are handled by the subproblem and will guarantee the validity of the solution that is decoded. Compared to Bender’s decomposition approach, the master problem, including the variables and the constraints, is not modified at each iteration. The subproblems solved at each iteration depend on the sub-solution generated at the master problem.

**Figure 1:**
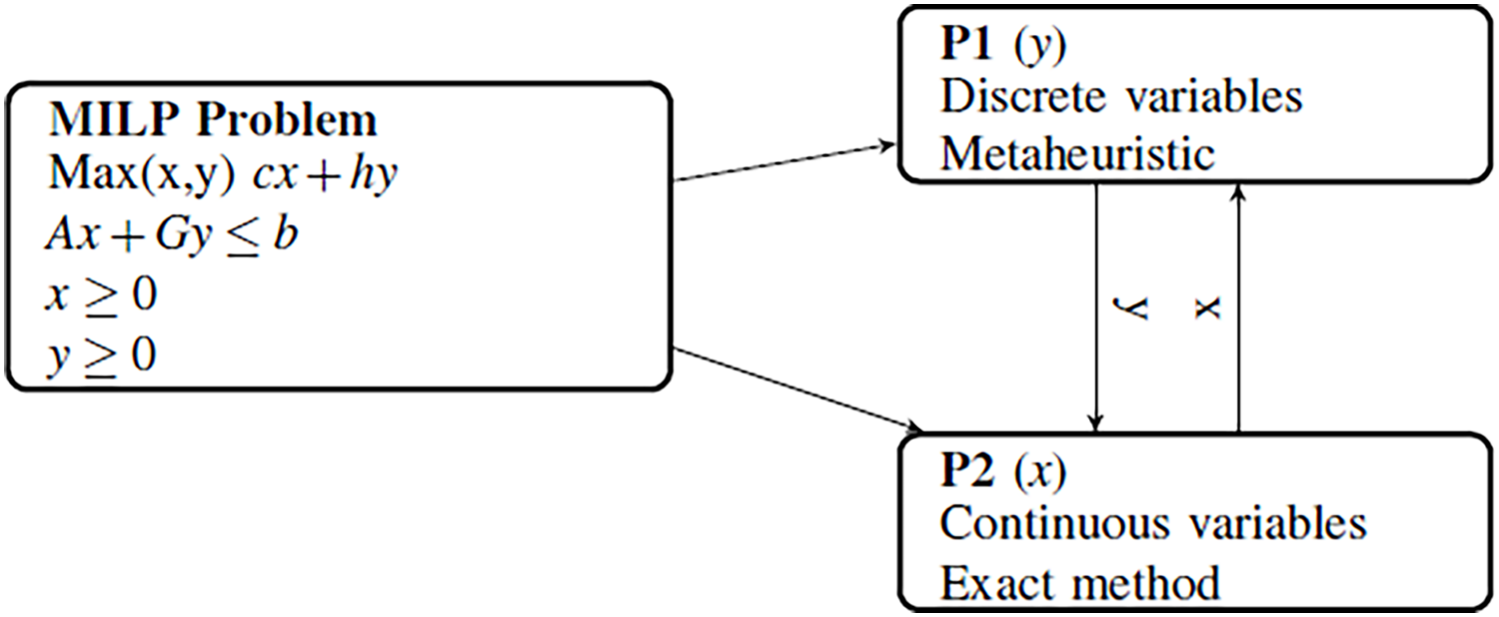
Variable decomposition of MILP problem into two subproblems.

Figure 1 shows how a general MILP problem is broken down into two hierarchical subproblems of different complexities:

- The master problem (P1) contains the discrete variables and is difficult to solve efficiently with an exact method. Then, metaheuristic approaches are more efficient to solve the master problem. In this paper, the hyper-heuristic methodology with a set of adaptive metaheuristics *H*_*i*_ (*i* = 1,…,*k*) is used to solve the master problem.
- The subproblem (P2), including the continuous variables, is a linear continuous problem (LP), and easy to solve using an exact linear solver. This subproblem decodes the incomplete solution of the master problem to obtain a complete solution for the problem.

## Matheuristic methodology

**Figure 2:**
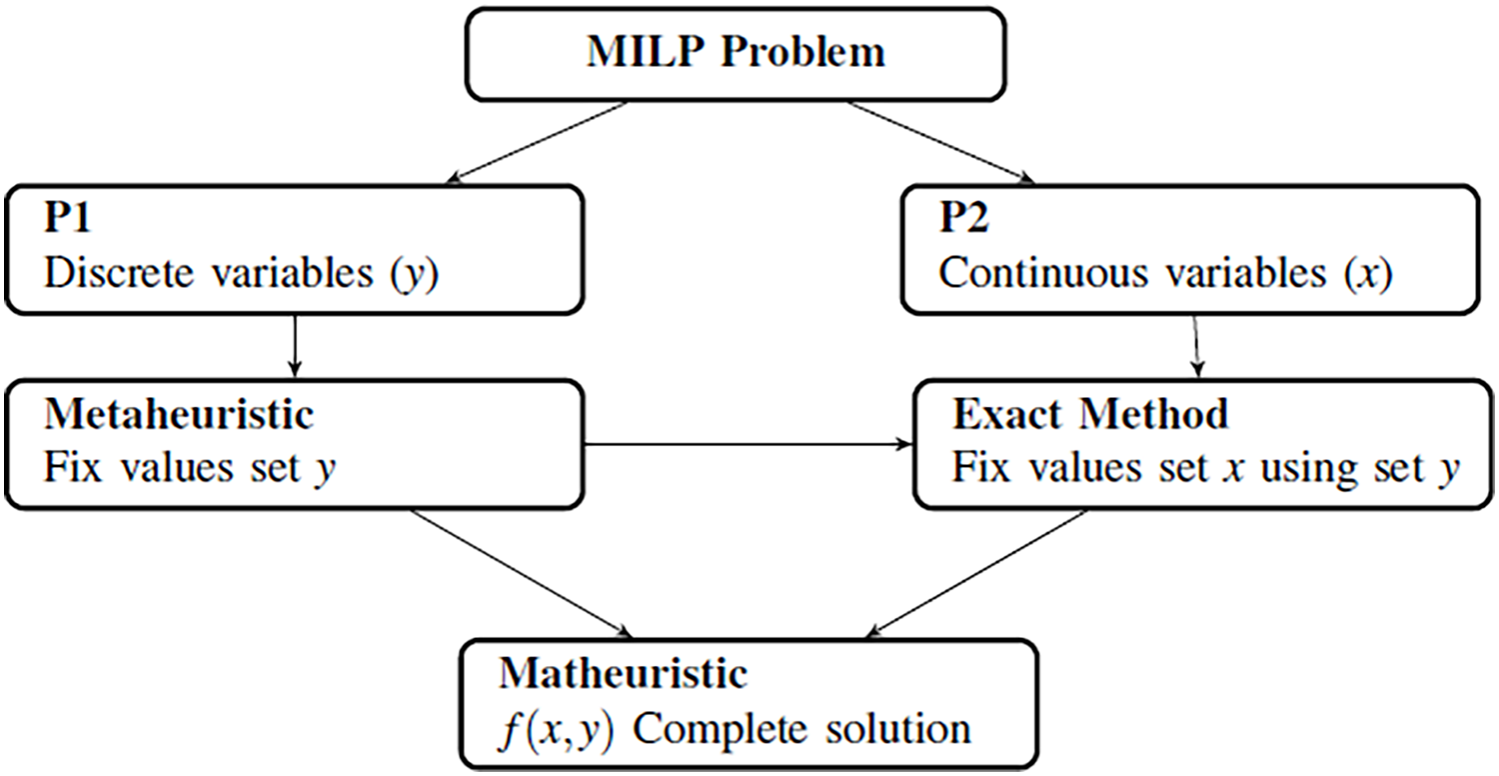
Generation of metaheuristics and structure of the algorithm.

The combination between metaheuristics and exact methods that is presented arises from the need to simplify mathematical models that are difficult to solve by any of these techniques. In this way, any mathematical model can be divided into different subproblems, taking the nature of its variables as the main criteria. In this paper, we propose to divide the model into two different subproblems where one of them must be a linear problem. In this case, it is easier to solve the linear problem using exact methods, and we can study the other subproblem using metaheuristic methods. Figure 2 shows how the decomposition is developed and how both methods collaborate.

The matheuristic algorithm is designed to be mainly used by population-based metaheuristics, in which a set of solutions are randomly generated and processed by different steps such as selection, recombination and replacement. However, single solution-based metaheuristics such as local search and tabu search can also be used.

After generating of the initial population using a metaheuristic, an exact method is employed to solve the subproblems generated. This method involves the use of relaxation or decomposition techniques of the mathematical model. Relaxation methods consist of relaxing a strict requirement in the target optimization problem (Sadykov et al., 2019). This method comprises disregarding the integrality constraints of an integer program and solving it using LP solvers. To do so, the metaheuristic generates the discrete variables (solving the subproblem P1), and provides this information to the exact method to fix values for the continuous ones (solving the subproblem P2). Figure 2 shows how the main problem is divided into two smaller problems.

All the functions incorporated in the metaheuristic are executed sequentially:

- **Initial population:** This step is used to generate the initial population (*Population*), fixing values for the discrete variables in P1.
- **Improvement:** Some of the feasible solutions (*TotalImprove*) are modified to improve the fitness value using the Variable neighborhood search algorithm proposed by (Mladenović & Hanse, 1997). The infeasible solutions are improved trying to transform them into feasible ones.
- **Selection:** We first sort the valid solutions, in decreasing order of fitness, followed by the invalid ones, which are ordered randomly. We then select a percentage of solutions to be used in the Crossover and Diversification function.
- **Crossover:** The algorithm includes a crossover function, which combines a certain number of pairs of solutions (*Combination*), which are chosen randomly from those previously selected.
- **Diversification:** A diversification based on edge recombination (ER) (Laporte, Potvin & Quilleret, 1997) is included, where the aim of diversification methods is to drive the search in new regions of the solution space. Here, the tabu search heuristic is periodically restarted with a new solution obtained through recombination of two elite solutions previously visited during the search (*Diversification*).

Depending on the metaheuristic selected, some internal functions may or may not be executed. For example, an EA does not use the improvements, or a GRASP does not employ the crossover function. At this point, a huge number of metaheuristics can be used to solve the problem. There are many options to determine the metaheuristic used, but in this article it has been decided to use a parameterized scheme, where certain parameters give value to the internal functionalities. This technique will be further detailed in “Experimental Results”. For each problem, the metaheuristic selected solves the problem P1. Then, the exact method is used to solve the problem P2 and obtain the initial complete population. Algorithm 1 shows how the algorithm works.

**Algorithm 1:** Matheuristic algorithm.

**REQUIRE:** MILP problem (*x*,*y*)
** ENSURE:** Best solution [*max f*(*x*,*y*)]
1 *//The selected metaheuristic could be that proposed in section 4 or another from the literature;* 2 Fix the metaheuristic parameters (*Population*, *Combination*, *TotalImprove*, *Diversification*)
3 *//Create S set of solutions;*
4 **for** *j* = 1 **to** *Population* **do**
5 Fix discrete variables *y*_*j*_ of problem *P*1;
6 Obtain continuous variables *x*_*j*_ solving *P*2 using the exact method;
7 $S \leftarrow [Solutio{n_j}: = ({y_j},{x_j})]$;
8 **if** *Solution*_*j*_ *is not feasible* **then**
9 Improve *Solution*_*j*_ using the Variable neighborhood search algorithm (Mladenović & Hanse, 1997);
10 **end**
11 **end**
12 **do**
13 //*Crossover SS subset of S such as |SS| > *1;
14 **for ***w* = 1 **to** *Combination* **do**
**15 **Parents $\leftarrow *RandomSelect*(*s*_1_ and *s*_2_ from *S*);
16 ${y_w} \leftarrow$ Crossover(*y*_1_ from *s*_1_, *y*_2_ from *s*_2_);
17 ${x_w} \leftarrow$ *ExactMethod*(P2,*y*_*w*_);
18 *s*_*w*_: = (*y*_*w*_,*x*_*w*_);
19 **if ***Fitness*(*s*_*w*_) > *Fitness*(*s*_1_) or *Fitness*(*s*_*w*_) > *Fitness*(*s*_2_) **then**
20 $SS \leftarrow {s_w}$;
21 **end**
22 **end**
23 *//Improve SSI subset of SS*;
24 **for ***w* = 1 **to** *TotalImprove* **do**
25 Select *s*_*w*_ ∈ *SS* randomly;
26 **REPEAT:;**
27 Modify *y*_*w*_ using the best neighbourhood algorithm and obtain *x*_*w*_ solving *P*2 using the exact method;
28 **UNTIL** *Fitness*(*s*_*w*_) increase or achieve *EndConditions*;
29 **end**
30 *//Diversify SSD subset of SSI*;
31 **for** *w* = 1 **to** *Diversification* **do**
32 Select *s*_*w*_ ∈ *SSI* randomly ;
33 Modify randomly *y*_*w*_ of *s*_*w*_;
34 Obtain *x*_*w*_ solving *P*2 using the exact method;
35 **end**
36 Include *SSD* in *S*
37 **while** *not EndCondition*;
38 ;
39 $BestSolutio{n_k} \leftarrow$ *s* ∈ *S* such as *Fitness*(*s*) ≥ *Fitness*(*w*) ∀ *w* ∈ *S*;

Of paramount importance is the handling of the constraints in the proposed decomposition methodology. The feasibility of the solutions strongly depends on the values obtained in the discrete variables of problem P1. That is because the constraints in the problem P2 are created using the values of the discrete variables. Therefore, the solutions of the problem P2 can be feasible or infeasible. A linear program is infeasible if there is no solution that satisfies all of the constraints at the same time. We evaluated and classified the infeasible solutions generated by the exact method by assigning them a value based on certain parameters of the exact method. This parameter relates to the number of restrictions not met by these solutions, and is modeled using a numeric value. We assign this fitness penalty-based value to infeasible solutions. When this value is close to 0 it means that the solution is close to being feasible, and implies that it needs fewer changes than other infeasible ones.

From the initial population, a number of elements from both groups are selected (feasible and infeasible solutions) and used to generate new solutions.

We first sort the valid solutions, in decreasing order of fitness, followed by the invalid ones, which are ordered randomly. We select a percentage of solutions to be combined and mutated. We select the best solutions (with the highest fitness) from the valid set while the solutions from the invalid set are selected randomly.

The algorithm includes a combination function that combines pairs of solutions, which are randomly chosen from those selected previously. The pair of solutions must belong to the same group, whereby valid solutions are combined with valid solutions and invalid solutions with invalid ones. These combinations generate new solutions that inherit some characteristics from their parents, and, for all the combinations, the algorithm only uses the discrete variables from P1. The remaining the variables are obtained by solving P1 using the exact method. To execute these combinations, a multi-point crossover operator was developed which generates an offspring by copying its genes from the parents, and which are chosen according to a randomly constructed crossover mask. We use this mask, which contains ones and zeros randomly generated with the same probability to generate new discrete variables. We determine the selected values from each of the two solutions by the mask in each position, taking the value from the first solution if there is 1 in the mask, or, if not, from the second one.

We also evaluated and improved all these new generated solutions in order to maximize the number of feasible solutions. Those steps of the algorithm are repeated a given number of times. Algorithm 1 is a schematic representation of the main matheuristic algorithm. This algorithm defines the extent to which the metaheuristic and the exact method are involved.

## Hyper-matheuristic methodology

In this work, a hyper-matheuristic framework is developed to generalize the matheuristic scheme proposed in González et al. (2017). We develop a hyperheuristic method on top of the matheuristic to find the best Metaheuristic in terms of fitness (optimal solution). For that, a set of metaheuristics (*H*) is created. In each iteration, the metaheuristic used in the matheuristic (*H*_*i*_) is updated in an adaptive way, obtaining new possible solutions and time values.

**Figure 3:**
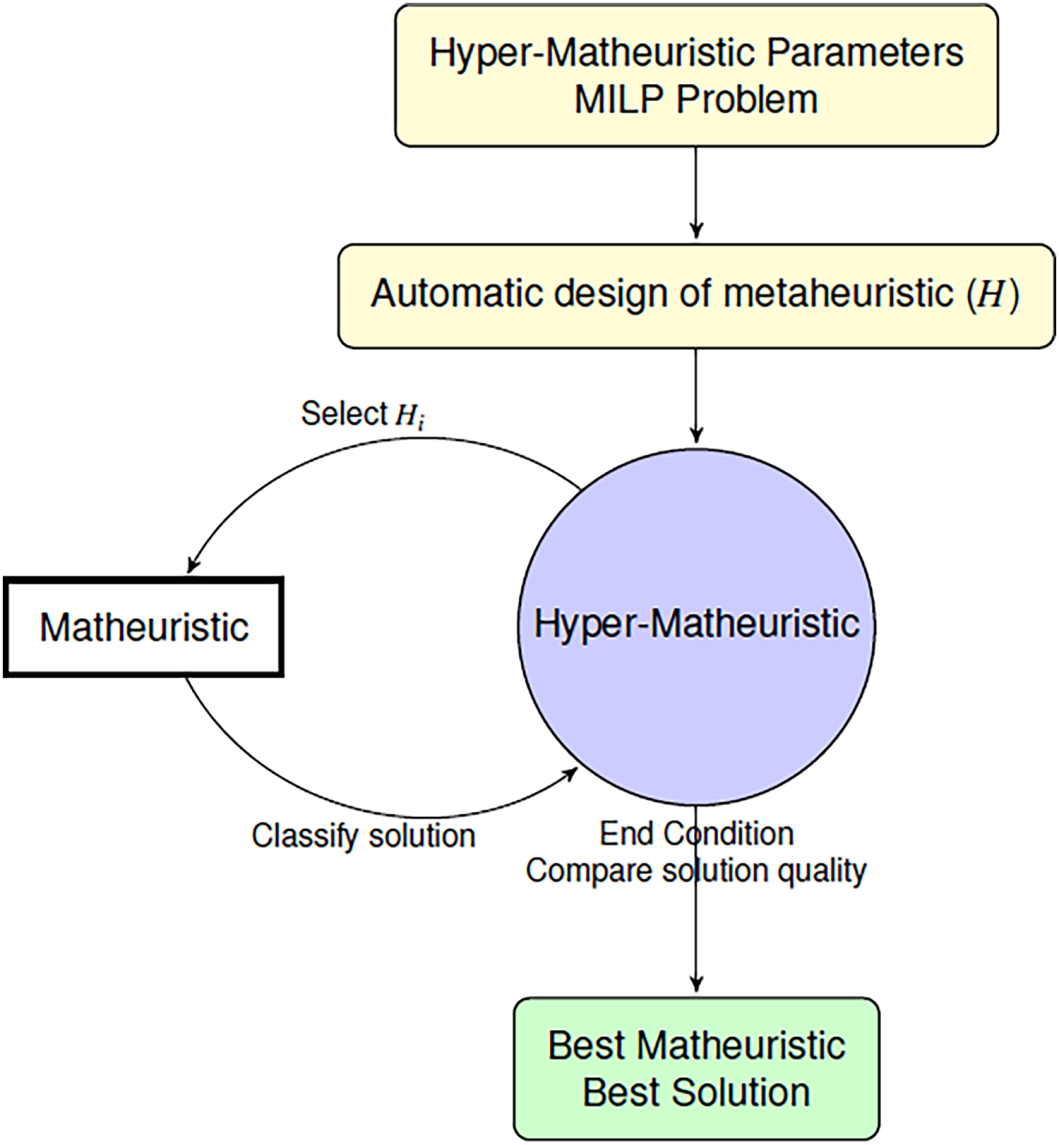
Algorithm to find the best matheuristic using the hyper-matheuristic method.

We propose a hyper-matheuristic algorithm to find the best suited metaheuristics, that combined with an exact method, generates the best solution for each problem in the shortest time possible (Fig. 3). This algorithm searches the whole metaheuristic space to find the best design. Then, two levels of metaheuristics are developed. One of them uses the objective function for each problem to evaluate the solutions (matheuristic), and the other one uses the average of all the objective values obtained and the time used to evaluate all the metaheuristics used (hyper-matheuristic).

The next subsections describe how the hyper-matheuristic framework works. First, the parameterized scheme to generate all the metaheuristics is introduced. Then, the hyper-matheuristic methodology is detailed.

### A parameterized scheme of metaheuristics

This scheme is included in the hyper-matheuristic and offers the possibility of generating and analyzing a large number of combinations between different metaheuristics. Depending on the problem evaluated, there is a large number of metaheuristic algorithms that can obtain good solutions. The objective of the scheme is to offer the possibility of using different metaheuristics for each problem, as well as being able to generate hybrid metaheuristics. This scheme is included in the work so as not to particularize a complete metaheuristic, but to use its most interesting functionalities. According to certain parameters, the parameterized scheme is able to generate hybrid metaheuristics that shares information from general schemes, like EA, GRASP or SS. This scheme has the possibility of designing a large number of metaheuristics in a general way, varying the value of all the parameters inside.

In this work, the main search components used for the design of a metaheuristic are: Initialize (*i.e*. initialization of the population), Improvements, Selection, Combinations (*e.g*. crossover) and Stopping Criteria. For each search component, several parameters are included. Table 1 summarizes the parameters used to generate some well-known and diverse sets of metaheuristics automatically.

**Table 1:** Parameters used in each basic function of the parameterized metaheuristic scheme.

Function	Parameters	Description
Initialize	INEIni	Initial number of elements
	FNEIni	Final number of elements selected for the iterations
	PEIIni	Percentage of elements to improve in the initialization
	IIEIni	Intensification of the improvement in the initialization
End condition	MNIEnd	Maximum number of iterations
	NIREnd	Maximum number of iterations without improving
Selection	NBESel	Number of best feasible solutions selected
	NWESel	Number of infeasible solutions selected
Combination	PBBCom	Number of combinations between feasible solutions
	PWWCom	Number of combinations between infeasible solutions
Improve	PEIImp	Percentage of crossover elements to be improved
	IIEImp	Intensification of the improvement
	PEDImp	Percentage of elements to diversify
	IIDImp	Intensification of the improvement to diversify elements

A parameterized scheme was already employed and tested in Almeida et al. (2013), where other parameters such as combinations between feasible and infeasible solutions were examined. The number and meaning of the parameters would vary if other basic metaheuristics were evaluated or if the basic functions were executed differently, but the parameters considered here allow us to automatically generate and experiment with different metaheuristics and combinations of them in order to improve the results obtained.

A large number of combinations can be considered simply by selecting different values for the considered parameters. The best metaheuristic with the parameterized scheme can be obtained by generating all the possible combinations of the parameters and by applying them to some small training set of problem instances. In this way, the generated combination of the various metaheuristics, given by the values of the parameters, is that which gives the best results in terms of the training set and can be deemed to be a satisfactory metaheuristic for the problem under consideration. There are many possible combinations of the parameters in the parameterized metaheuristic scheme, obtaining the best metaheuristic for the training set is an expensive optimization problem, and therefore suitable for the hyper-matheuristic.

### Hyper-matheuristic

To obtain the best metaheuristic that provides the best objective quality in less search time, a metaheuristic has been developed at a higher level called hyper-matheuristic. This new metaheuristic is developed to generate, evaluate and improve different types of metaheuristics (set *H*_*i*_). The developed hyper-matheuristic makes it possible to design a set of metaheuristics *H*_*i*_ automatically. Then, the hyper-matheuristic is a metaheuristic included on top of the matheuristic algorithm that is able to select the best value of the parameters in the Parameterized Scheme, with the aim of designing an efficient metaheuristic that, combined with an exact method, provides the best objetive funcion value. The generated metaheuristics depend on the value of the parameters in the parameterized scheme. Depending on the solution quality obtained for each problem and the search time used, the hyper-matheuristic is able to adapt itself modifying the next metaheuristic to improve on the previous one. For that, this algorithm saves all the information about all metaheuristics in *H*_*i*_, thus it learns every step online. In order to have an initial reference, the EA, GRASP and SS methods have been established as diverse metaheuristic prototypes, so that all the metaheuristics generated by the hyper-matheuristic will have certain functionalities of those general metaheuristic frameworks. To determine the number of metaheuristics evaluated for each problem, as well as the number of applied improvements and changes, a certain number of parameters have been designed to control these aspects:

- NIM_EA: Number of initial metaheuristics generated from evolutionary algorithms (EA).
- NIM_SS: Number of initial metaheuristics generated from scatter search (SS).
- NIM_GRASP: Number of initial metaheuristics generated from greedy randomized adaptive search procedure (GRASP).
- NIM: Total number of initial metaheuristics
- (NIM_EA+NIM_SS+NIM_GRASP).
- NFM: Number of new metaheuristics created by recombination.

First, a given number of metaheuristics (NIM) based on EA, GRASP and SS are generated in the reference set. In order to achieve the desired metaheuristic functionalities, values are assigned to the parameters in Table 1, establishing values between 0 and a preset limit that has been evaluated during the experiments. The established limits for each parameter can be modified in each execution to study the variances in the solution quality and the search time obtained. In each iteration, the MILP problem is solved using a metaheuristic included in *H*_*i*_, providing values of solution quality and search time.

When the initial population is created, all the created metaheuristics are selected to be improved. Several improved functions are developed to modify the metaheuristic parameters, increasing the parameters associated to some functionalities such as the initial population, improvements or crossovers while decreasing others at the same time. With these improved functions, the metaheuristics are evaluated and compared depending on their functionalities. The new improved metaheuristics are added to the reference set. With this reference set, the crossover function is executed. Hence, new metaheuristics are created, where their parameters are selected from the metaheuristics in the reference set. The parameters from the metaheuristics of a better quality have a higher probability of being selected. Algorithm 2 shows how the proposed hyper-matheuristic methodology works.

**Algorithm 2:** Hyper-matheuristic algorithm.

**REQUIRE:** Hyper-matheuristic parameters, MILP Problem
**ENSURE:** *Hi*, Best metaheuristic.
1 *Fix limits for all the parameters in the scheme (upper and lower bound);*
2 **for** *i* = 1 **to** *NIM* **do**
3 *Generate matheuristic* H_i_;
4 **End**
5 **for** *i* = 1 **to** *NIM* **do**
6 *Solve problem using the matheuristic in H*_*i*_;
7 **for** *k* = 1 **to** *i* **do**
8 **if** (*H*_*i*_ *solution quality*) < (*H*_*k*_ *solution quality*) **then**
9 *Compare parameters between the metaheuristics*;
10 *Improve the matheuristic H*_*i*_ *modifying the parameters with more differences*;
11 **end **
12 **end**
13 **end**
14 **for** *i* = *NIM* **to** *NIM* + *NFM* **do**
15 *Select two matheuristics randomly from those generated in step 3*;
16 *Combine their parameters to create a new metaheuristic*;
17 *Execute steps from line 6 to 12*;
18 **end**
19 *Classify all the solutions by quality and time;*
20 *Select the best matheuristic (which maximize fitness/time), and save parameters;*

## Experimental results

We performed various experiments to analyze the effectiveness of the hyper-matheuristic methodology for a given MILP problem coming from the DEA literature. To be specific, the aim of the solved problem is to ascertain the technical efficiency of a set of n firms (in general, a set of Decision Making Units - DMUs), which use *m* inputs to generate *s* outputs. To that end, we will apply a well-known non-parametric technique, called Data Envelopment Analysis (DEA) (Charnes, Cooper & Rhodes, 1978). The MILP problem to be solved must be executed for each DMU of the sample of observations. Specifically, we focus our analysis on the Slacks-Based Measure developed in Aparicio, Ruiz & Sirvent (2007), since it must be computed by the MILP. With regard to the data, in our simulations the *m* inputs and *s* outputs of each of the *n* DMUs are generated randomly but bearing in mind that the well-known Cobb-Douglas function (Cobb & Douglas, 1928) is the function governing the production situation.

Let us assume that data on *m* inputs and *s* outputs for *n* DMUs are observed. For the *j*th DMU these are represented by *z*_*ij*_ ≥ 0, *i* = 1,…,*m* and *q*_*rj*_ ≥ 0, *r* = 1,…,*s*. The DEA model that should be solved as follows:

(3) $$\matrix{ {\max \left\{ {{\beta _k} - \displaystyle{1 \over m}\sum\limits_{i = 1}^m \displaystyle{{t_{ik}^ - } \over {{z_{ik}}}}} \right\}} \cr {\rm s.t.} \cr {\matrix{ {{\beta _k} + \displaystyle{1 \over s}\sum\limits_{r = 1}^s \displaystyle{{t_{rk}^ + } \over {{q_{rk}}}} \le 1} & {} & {(c.1)} \cr { - {\beta _k} - \displaystyle{1 \over s}\sum\limits_{r = 1}^s \displaystyle{{t_{rk}^ + } \over {{q_{rk}}}} \le - 1} & {} & {(c.2)} \cr { - {\beta _k}{z_{ik}} + \sum\limits_{j = 1}^n {\alpha _{jk}}{x_{ij}} + t_{ik}^ - \le 0} & {\forall i = 1,\ldots,m} & {(c.3)} \cr {{\beta _k}{z_{ik}} - \sum\limits_{j = 1}^n {\alpha _{jk}}{x_{ij}} - t_{ik}^ - \le 0} & {\forall i = 1,\ldots ,m} & {(c.4)} \cr { - {\beta _k}{q_{rk}} + \sum\limits_{j = 1}^n {\alpha _{jk}}{y_{rj}} - t_{rk}^ + \le 0} & {\forall r = 1,\ldots ,s} & {(c.5)} \cr {{\beta _k}{q_{rk}} - \sum\limits_{j = 1}^n {\alpha _{jk}}{y_{rj}} + t_{rk}^ + \le 0} & {\forall r = 1,\ldots ,s} & {(c.6)} \cr { - \sum\limits_{i = 1}^m {\nu _{ik}}{z_{ij}} + \sum\limits_{r = 1}^s {\mu _{rk}}{q_{rj}} + {d_{jk}} \le 0} & {\forall j = 1,\ldots ,n} & {(c.7)} \cr {\sum\limits_{i = 1}^m {\nu _{ik}}{z_{ij}} - \sum\limits_{r = 1}^s {\mu _{rk}}{q_{rj}} - {d_{jk}} \le 0} & {\forall j = 1,\ldots ,n} & {(c.8)} \cr { - {\nu _{ik}} \le - 1} & {\forall i = 1,\ldots ,m} & {(c.5)} \cr { - {\mu _{rk}} \le - 1} & {\forall r = 1,\ldots ,s} & {(c.6)} \cr { - {d_{jk}} \le - M{b_{jk}}} & {\forall j = 1,\ldots ,n} & {(c.7)} \cr {{\alpha _{jk}} \le M(1 - {b_{jk}})} & {\forall j = 1,\ldots ,n} & {(c.8)} \cr {{b_{jk}} = 0,1} & {\forall j = 1,\ldots ,n} & {(c.9)} \cr { - {\beta _k} \le 0} & {} & {(c.10)} \cr { - t_{ik}^ - \le 0} & {\forall i = 1,\ldots ,m} & {(c.11)} \cr { - t_{rk}^ + \le 0} & {\forall r = 1,\ldots ,s} & {(c.12)} \cr { - {d_{jk}} \le 0} & {\forall j = 1,\ldots ,n} & {(c.13)} \cr { - {\alpha _{jk}} \le 0} & {\forall j = 1,\ldots ,n} & {(c.14)} \cr } } \cr }$$

where *M* is a large, positive number. For this specific MILP problem, the vector of continuous variables *x* consists of (*β*_*k*_, $t_{ik}^ -$, $t_{rk}^ +$, *d*_*jk*_ and *α*_*jk*_), while the vector of integer variables consists exclusively of *b*_*jk*_.

The number of feasible solutions obtained in the initial population using the algorithm put forward in this paper is studied for various population sizes and optimization methods. In the first experiment, the methods proposed above for solving the main problem are evaluated, comparing the results of solving the problem globally (heuristic) with those obtained by applying the decomposition of the problem (matheuristic). In addition, a hybrid method in which both techniques are executed in a cooperative way is added. After that, the obtained solution quality with basic metaheuristics with parameters similiar of those of EA, GRASP or SS is compared with hybrid metaheuristics generated automatically with a hyper-matheuristic using the parameterized scheme shown in “A Parameterized Scheme of Metaheuristics”. The hyper-matheuristic is trained with many instances to obtain a satisfactory metaheuristic for any problem size.

Finally, we compare the solution quality and the execution time obtained using the satisfactory metaheuristic with those obtained using other metaheuristics. For all the experiments, we use the IBM ILOG CPLEX Optimization Studio (CPLEX). The experiments are executed in a parallel NUMA node with 4 Intel hexa-core Nehalem-EX EC E7530, with 24 cores, at 1.87 GHz and 32 GB of RAM. The environment used to run the application is a Centos 8 operating system, using C code to develop the algorithm. Additionally, the Intel C++ Compiler was used and the Intel MKL Libraries were included.

### MILP-decomposition vs global problem solving

To evaluate the proposed decomposition strategy, several experiments have been carried out, in which the focus is made on each of the algorithm stages. First, the generation of the initial population was evaluated. Then the algorithm was executed using several generation methods: heuristic method and matheuristic method. The heuristic method used is proposed in González et al. (2015) where a problem dependent algorithm is developed. This heuristic does not use a decomposition variable method. Therefore, it is a good option to compare it with the matheuristic algorithm used in this paper. The hybrid method is a combination of the proposed heuristic in González et al. (2015) and exact methods, where the number of problems to evaluate (DMUs) is divided in two groups. One of the groups is solved by the exact method, and the other is solved by the heuristic method. In this experiment, the parameters have been fixed, being the same in all the methods used. The population size (INEIni) is set by default to 100. In addition, another implementation of the exact method has been included, where the number of initial solutions (INEIni) has been increased to 1,000 to analyze the impact of this parameter.

Table 2 shows the average of the objective values and the percentage of feasible solutions obtained in the Initialization step of Algorithm 1, according to the method used and using several values for the initial population. The experiment shows that it is non-trivial to obtain feasible solutions using the matheuristic method, because the search space is huge, and the metaheuristic needs to make a great effort to obtain satisfactory values for the discrete variables. When the initial population grows, the number of feasible solutions increases (comparison between matheuristic with population size of 100 and 1,000). Moreover, the solution quality value greatly depends on the initial population. When the matheuristic is used with a population size of 1,000 solutions, the value of the fitness of the obtained solutions using the matheuristic improves compared to the other methods. Thus, it can be observed that, for the same initial population value, the heuristic method obtains a higher number of feasible solutions but with a lower quality. So, obtaining feasible solutions is a really difficult task in terms of the solution space. Metaheuristics need a huge effort to find feasible solutions in the set of discrete variables that satisfy all the constraints in the continuous variables. Additionally, the quality of the solutions suggests that only a few solutions are needed to obtain the optimal one. Now, it is the moment to evaluate the complete matheuristic algorithm and compare fitness and the amount of computational time required.

**Table 2:** Average of percentage of feasible solutions and fitness solution obtained at the initialization step for the different optimization methods and varying the INEIni parameter.

Size			Matheuristic 100		Heuristic method		Hybrid method		Matheuristic (1,000)	
*m*	*n*	*s*	% val.	Fitness	% val.	Fitness	% val.	Fitness	% val.	Fitness
2	100	1	18_5.16_	0.7605	94.96_6.47_	0.685	56.946_8.24_	0.413	6.032_1.02_	0.7609
3	50	1	1.32_1.61_	0.4748	78.85_5.90_	0.777	43.441_7.82_	0.5661	1.221_0.77_	0.7974
3	100	1	0.55_1.37_	0.2713	66.12_5.17_	0.6171	32.539_5.23_	0.48	0.758_0.52_	0.6662
4	100	1	0.29_0.54_	0.1544	60.71_6.72_	0.588	25.015_6.12_	0.414	0.305_0.82_	0.5515
5	100	1	0.33_0.81_	0.1974	58.66_8.36_	0.501	39.485_9.08_	0.398	0.371_0.15_	0.5164

### Matheuristic in the parameterized scheme of metaheuristics

**Figure 4:**
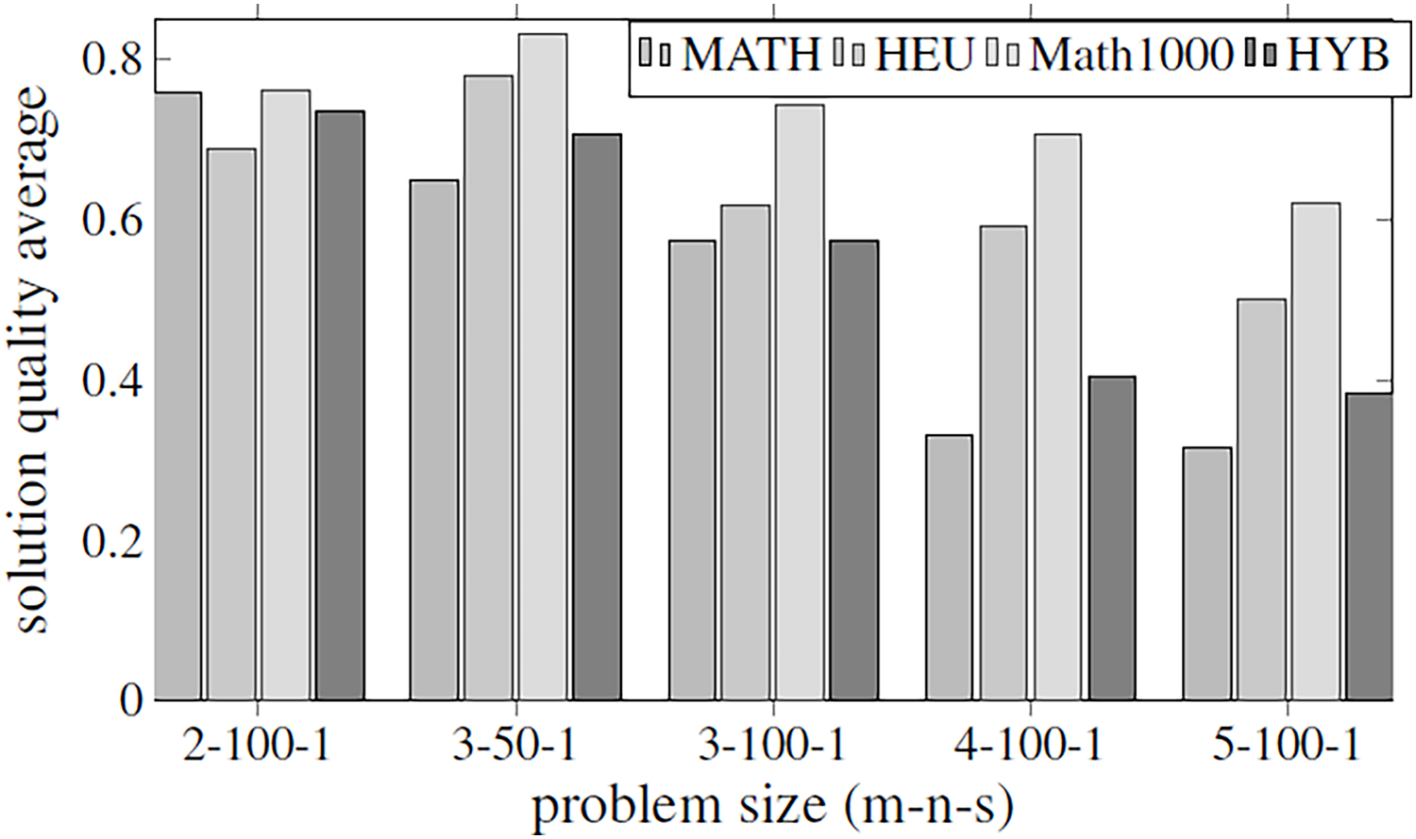
Objective values obtained by the methods proposed in Table 2 using all the parameters of Table 1. The methods compared are matheuristic (MATH), heuristic (HEU), hybrid method (HYB) and matheuristic using a population size of 1,000 (Math1000).

**Figure 5:**
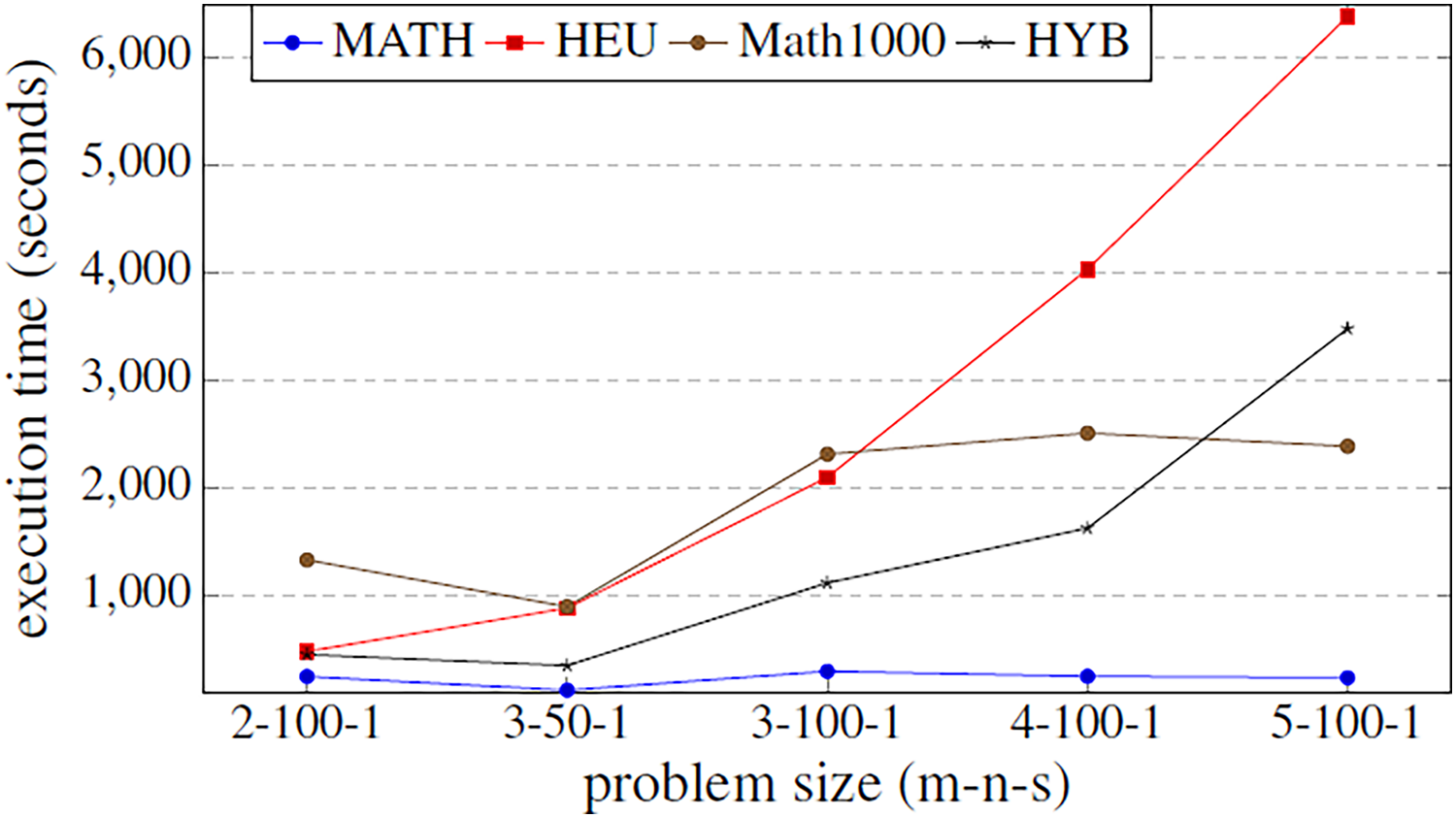
Comparison of the execution time function (in seconds) of the problem size. The methods compared are matheuristic (MATH), heuristic (HEU), hybrid method (HYB) and matheuristic 1,000 (Math1000).

Before executing the complete algorithm and developing the best-found hyper-matheuristic, the algorithm has been evaluated with fixed parametrized scheme values. The metaheuristics used for the two methods are configured to be as versatile as possible (with all internal functions), using low values to improve the execution time. In order to make fair time comparisons, the values of the various parameters for the different optimization methods are set to the same values: INEIni = 100, FNEINI = 50, IIEIni = 10, PEIIni = 10 NBESel = 15, NWESel = 15, PBBCom = 25, PWWCom = 25, PEEImp = 10, IIEImp = 5, PEDImp = 5, IIDImp = 5, NIREnd = 5, MNIEnd = 10. The solution quality is shown in the Fig. 4, and the execution time in Fig. 5.

The results obtained show that there is a high correlation between the execution time and the quality of the obtained solutions. It can be observed that using the matheuristic with a low number of initial solutions (INEIni), the solution quality obtained is the lowest, but the solution is found in the least time. On the other hand, it is observed that the matheuristic is much faster than the heuristic method (see Fig. 5). However, the solution quality obtained by the heuristic method for these low initial population values is better than those obtained by the matheuristic. This leads to the conclusion that, in order to obtain an efficient hyper-matheuristic method in the following steps, the heuristic method must be discarded. The heuristic method would only be optimal for small problem sizes. Since we want to make an algorithm independent of the type and size of problem, it was preferred not to use this method and only design the hyper-matheuristic including the matheuristic as such. This experiment concludes with the comparison between the fitness obtained by the feasible solution and the computational costs required. The figures explain that the initial population is a critical parameter, the computational time during which the size of the problem grows being a great limitation. Both values (fitness and time) must be evaluated and taken into account in the following experiments.

### Hyper-matheuristic vs general metaheuristics

To develop the hyper-matheuristic methodology, some general metaheuristics are used. The automatic design of the best hyper-matheuristic within this space of algorithms is the main issue. All the metaheuristics generated by the hyper-matheuristics will inherit some characteristics from the general metaheuristics, and will combine others. The proposed metaheuristics to be executed in the experiments are shown in Table 3. The lowest and the highest values of the parameters of these metaheuristics are used to limit the values of all the parameters within the hyper-matheuristic. Then, all the metaheuristics will generated using these limits.

**Table 3:** Values of the parameters for the three basic metaheuristics considered and the hyper-matheuristic limits.

Metaheuristic	IINEIni	FNEIni	PEIIni	IIEIni	NBESel	NWESel	PBBCom
EA	300	150	0	0	100	0	50
GR	500	1	100	20	0	0	0
SS	100	50	50	5	25	25	25
Hyper	100/500	1/150	0/100	0/20	0/100	0/25	0/50
Metaheuristic	PWWCom	PEIImp	IIEImp	PEDImp	IDEImp	MNIEnd	NIREnd
EA	0	0	0	10	10	10	5
GR	0	0	0	0	0	10	5
SS	25	50	10	0	0	10	5
Hyper	0/25	0/50	0/10	0/10	0/10	0/10	0/5

**Figure 6:**
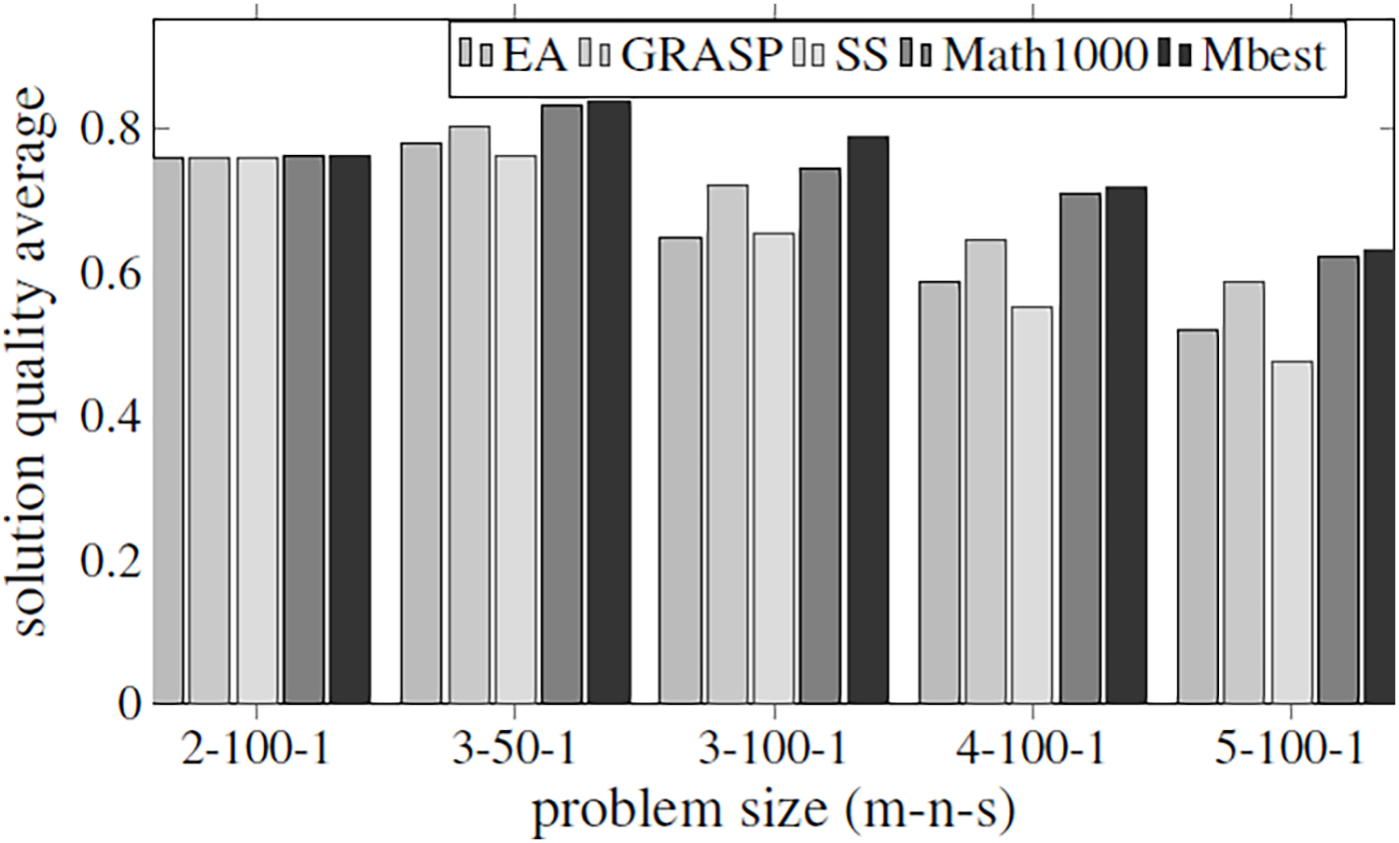
Fitness values obtained by the three basic metaheuristics, an exact method and the hyper-matheuristic (Mbest), varying the problem size.

The aim of this experiment is to be able to set the values for all parameters using them as default values for any problem size. A specific problem has been established (3/50/1) to train the hyper-matheuristic and get a good configuration. This training has been performed by running the hyper-matheuristic for this problem with 100 different metaheuristics and 100 combinations of these, where each evaluated metaheuristic has been tested 10 times for each model, obtaining the average value for each metaheuristic. At the end of the experiment, the best average quality of the 200 parameter settings (which means 200 different metaheuristics) has been obtained. For the solution quality evaluation of each metaheuristic, the average quality of all executions and DMUs has been taken into account, as well as the average time required to terminate the search. In conclusion, a ratio between solution quality and search time (*fitness*/*time*) is used to rank the metaheuristics according to the solution quality obtained and the search time. In Fig. 6, all the obtained qualities are compared between the three general metaheuristics proposed (EA, GRASP and SS), the best metaheuristic obtained by the hyper-matheuristic (Mbest) and the matheuristic method with initial population of 1,000 solutions (Math1000).

**Figure 7:**
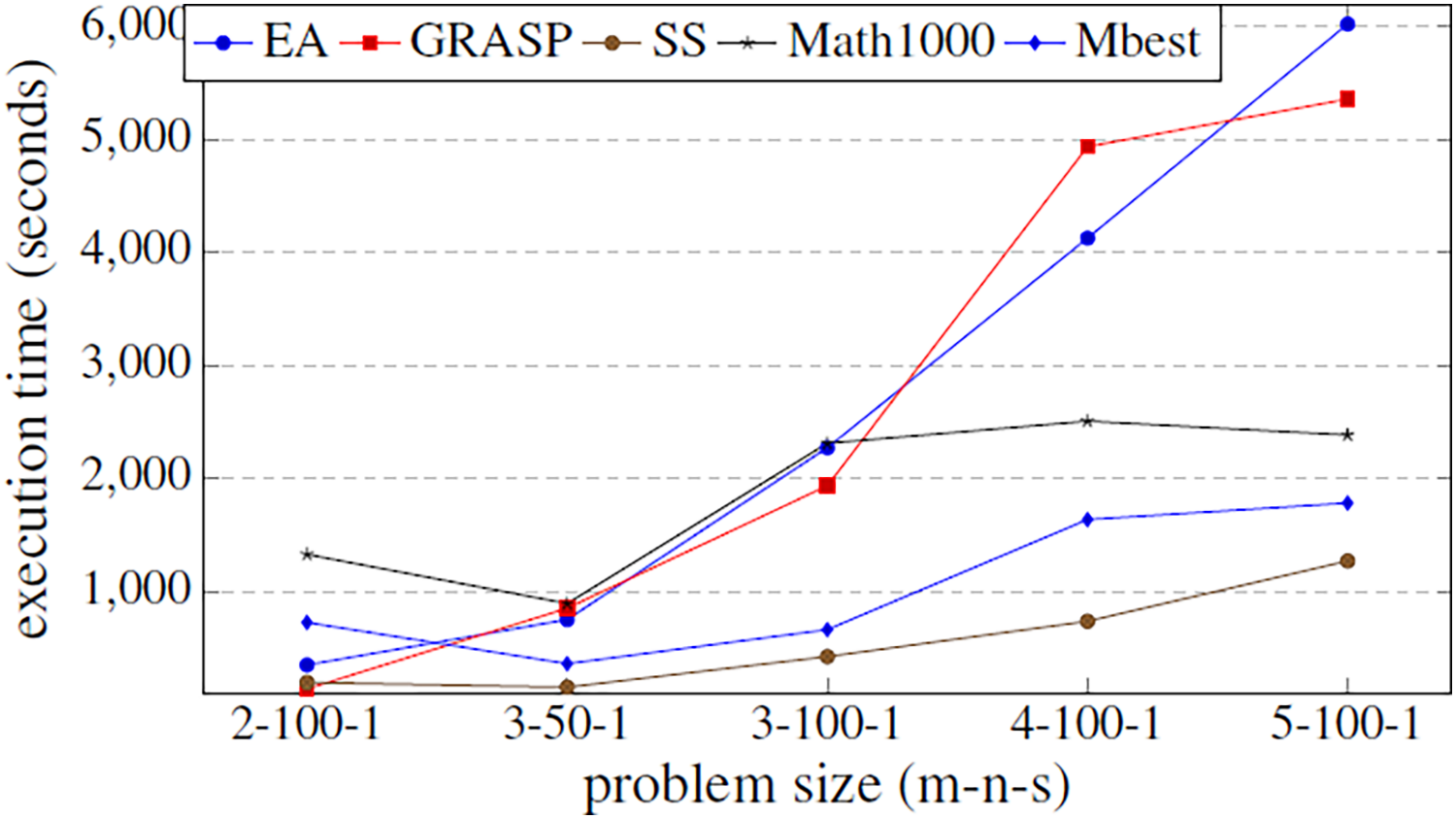
Comparison of the execution time (in seconds) between the three basic metaheuristics, the exact method and the hyper-matheuristic (Mbest) function of the problem size.

Figure 6 shows that the values obtained for the parameters in the hyper-matheuristics always improve the solution quality obtained with the other metaheuristics. The results obtained (Table 4) show that all the functions developed are used, but they do not need high values. It can be observed that for the three proposed metaheuristics, the GRASP strategy obtains better fitness solution. On the other hand, SS has the lowest initial population solutions and then lower probability of obtaining the best solution, despite incorporating all the functionalities. The fitness solution obtained with the Mbest is quite similar to the quality obtained by the matheuristic using the value of 1,000 in the initial solution set, but if the execution time is compared (Fig. 7), the Mbest is always faster than the matheuristic with the default parameter values used in the previous experiments. We can conclude that the trained hyper-matheuristic is able to obtain a good configuration of parameters, improving the quality and time for any problem size.

**Table 4:** Values of the parameters for the best metaheuristic (Mbest) found by the hyper-matheuristic in the matheuristic.

Metaheuristic	IINEIni	FNEIni	PEIIni	IIEIni	NBESel	NWESel	PBBCom
Hyper	310	61	13	17	12	10	22
Metaheuristic	PWWCom	PEIImp	IIEImp	PEDImp	IDEImp	MNIEnd	NIREnd
Hyper	25	12	8	3	7	6	5

### Bender’s decomposition evaluation

**Figure 8:**
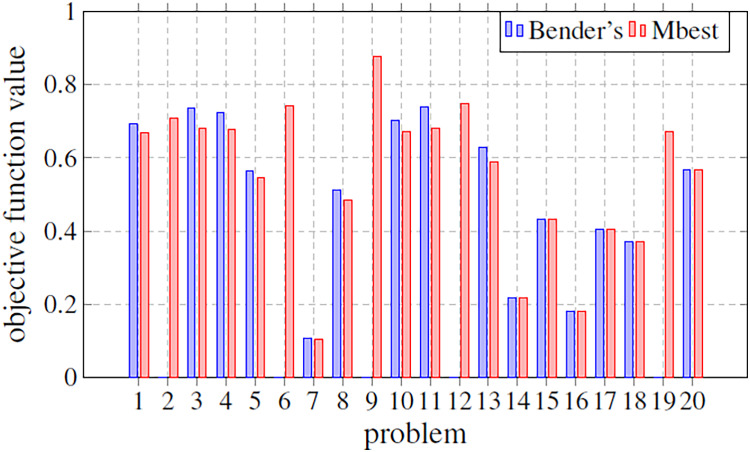
Comparison of the objective function value in several problems with a fixed size (*m* = 3; *s* = 1; *n* = 100). The methods compared are Bender’s Decomposition (Bender’s) and the best matheuristic found by the hyper-matheuristic (Mbest).

In this subsection, our approach and that based on solving the MILP problem by the optimizer and Bender’s decomposition are compared. For this, a last experiment was carried out, where based on a fixed size (*m* = 3, *s* = 1, *n* = 100), different problems generated have been evaluated obtaining, for both techniques, the optimal value of the objective function. Figure 8 shows a comparison between these two techniques (Bender’s and Mbest). In particular, it graphically illustrates the objective function value for 20 simulated problems by means of bars. The bar is not drawn if the corresponding technique does not find any solution. Something that only happens for Bender’s decomposition. Figure 8 shows how, when both techniques find an optimal solution, Bender’s and Mbest get very similar values. For some problems, Bender’s decomposition does not find any feasible solution. This contrasts with the Mbest technique, which is able to find solutions for all the cases studied. From this, it can be deduced that the proposed technique is valid for all the problems evaluated and that, in addition, it finds solutions very close to the optimum, since very similar results are obtained when comparing them with those obtained by an exact method through Bender’s decomposition. Therefore, we can see that the hyper-matheuristic introduced fulfils the proposed objective satisfactorily, being a complementary technique to those already known in the literature.

Our conclusions associated with the computational experience are limited by the type of optimization problem that was analyzed, within the context of Data Envelopment Analysis. Further evaluations of the new methodology on different types of optimization problems (such as packing, knapsack, inventory, *etc*.) would be needed to lead to more robust conclusions.

## Conclusions and future works

In this paper, we have developed an efficient decomposition strategy for MILP optimization problems in the context of Data Envelopment Analysis (DEA). We have developed a hierarchical decomposition based on the nature of the decision variables (continuous *vs* discrete) and the complexity of the subproblems. An incomplete encoding representing only discrete decision variables is explored by the metaheuristics. The encoding of solutions is completed for the continuous decision variables by solving a linear problem exactly.

This matheuristic framework has shown its validity in solving MILP problems in the framework of DEA. Moreover, we developed a hyper-matheuristic methodology on top of the parameterized metaheuristic scheme. It allows the automatic design and configuration of a flexible and generic template for population-based metaheuristics. Satisfactory results have been obtained in terms of solution quality and execution time. Other computational intelligence algorithms could be used to solve the problems, like the monarch butterfly optimization (MBO) (Wang, Deb & Cu, 2019) or the earthworm optimization algorithm (EWA) (Wang, Deb & Coelho, 2018).

One of the future research lines of this paper is to apply this hyper-matheuristic methodology to other real-life optimization problems formulated as MILP, such as unit commitment problems in power energy systems and demand side management in smart grids. Another perspective consists in the generalization of the proposed decomposition scheme for other families of optimization problems, in which only the continuous part of the problem is linear and easy to solve using an exact algorithm. Indeed, the most important feature of the proposed decomposition scheme is the complexity of the subproblems generated by metaheuristics and solved by exact algorithms. Another interesting perspective is to investigate the parallel design and implementation of the hyper-matheuristic methodology. Indeed, the proposed decomposition strategy is suitable to be deployed on heterogeneous parallel architectures composed of clusters of multiple cores and GPUs (Graphics Processing Units).

As a limitation, we point out that the proposed algorithm has been proved in only one kind of MILP problem, within the Data Envelopment Analysis field. Also, the algorithm developed in this paper is just applicable to MILP problems which include both discrete and continuous variables. An interesting future line of research would be to apply the new methodology to different specific types of optimization problems: packing, knapsack, inventory, production planning, location, resource allocation, routing or scheduling problems, to name but a few. This analysis would allow us to shed light on the adequacy of the new approach for solving very different optimization problems with varied structures. Another possible line of further research would consist of incorporating Bender’s decomposition to our approach to improve the computational time or, even consider Bender’s method as a new feature in the hyper-matheuristic.

## Supplemental Information

10.7717/peerj-cs.828/supp-1 Supplemental Information 1 Main application code. Contains all the main functions and the core of the algorithm.

10.7717/peerj-cs.828/supp-2 Supplemental Information 2 Bender experiment. This code includes the last experiment, where the Bender decomposition is executed under CPLEX and evaluated with our algorithm.

## Additional Information and Declarations

### Data Availability

The following information was supplied regarding data availability: The code is available in the Supplemental Files.
